# Health security needs a European health and care workforce strategy, and it needs it now

**DOI:** 10.1016/j.healthpol.2026.105597

**Published:** 2026-05

**Authors:** Ellen Kuhlmann, Tiago Correia, Katarzyna Czabanowska, Michelle Falkenbach, Marius-Ionuț Ungureanu, Matthias Wismar, Tomas Zapata

**Affiliations:** a Institute for Economics, Labour and Culture (IWAK), Goethe-University Frankfurt, Germany; b WHO Collaborating Center for Health Workforce Policies and Planning, Instituto de Higiene e Medicina Tropical, Universidade Nova de Lisboa, Lisbon, Portugal; c Global Health and Tropical Medicine, GHTM, Associate Laboratory in Translation and Innovation Towards Global Health, LA-REAL, Instituto de Higiene e Medicina Tropical, IHMT, Universidade Nova de Lisboa, UNL, Portugal; d Department of International Health, Care and Public Health Research Institute (CAPHRI), Maastricht University, Maastricht, the Netherlands; e WHO Collaborating Center for Public Health Leadership and Workforce Development, Maastricht University, Maastricht, the Netherlands; f European Observatory on Health Systems and Policies, Brussels, Belgium; g Department of Health Management and Policy, University of Michigan, Ann Abor MI, USA; h Department of Public Health, Faculty of Political, Administrative and Communication Sciences, Babeș-Bolyai University, Cluj-Napoca, Romania; i Center for Health Workforce Research and Policy, Faculty of Political, Administrative and Communication Sciences, Babeș-Bolyai University, Cluj-Napoca, Romania; j World Health Organization Regional Office for Europe, Copenhagen, Denmark

**Keywords:** Health security, Health and care workforce, Health workforce crisis strategy, Health workforce preparedness, European union strategy

## Abstract

Europe is currently facing novel security threats in many different areas, reinforcing the need for a well prepared and protected health and care workforce to ensure health system resilience and service provision for the population under conditions of a poly-crisis. However, the health and care workforce is weakened by persisting shortages, competency gaps and mismatches, and poor working and mental health conditions. Health and care workers are not prepared for yet another crisis and a systematic strategy is lacking. This policy commentary argues for health and care workforce preparedness and protection as a structural pillar and integral part of an emerging EU health and security landscape, calling for a coherent European Union strategy and highlighting capacities for implementation and co-benefits for democratic societies and economies. Key policy recommendations include: developing a coordinated EU strategy that is capable to protect, prepare and retain health and care workers; closing the competencies gaps to align preparedness for military aggression, cyberattacks, climate change, and new infectious diseases; investing in research and data spaces to strengthen evidence-based information and policy; creating governance structures and building on existing EU programs and budgets to freeing resources for the health and care workforce.

## 1 Introduction

Europe is currently facing novel and converging security threats across different areas, reinforcing the need for a well prepared and protected health and care workforce (HCWF) to ensure national security, health system resilience and service provision for the population under conditions of a poly-crisis. However, the HCWF remains weakened by persistent shortages, competency gaps and mismatches, and poor working and mental health conditions [[1], [2], [3], [4]]. Health and care workers (HCWs) are not prepared for yet another crisis. Russia’s war against Ukraine and growing aggression against Europe, violations of human rights and attacks against democratic states under the second Trump government in the United States [5], escalating cyber threats, and the accelerating impacts of climate change have converged into a poly-crisis affecting European health systems [6] and leaving the HCWs insufficiently protected.

Although Europe has strengthened collaboration for infectious diseases prevention [[7], [8], [9]] and health labour market policies [1,[10], [11], [12], [13], [14]], the European Union (EU) still lacks a comprehensive health security and workforce strategy. The lessons of the COVID-19 pandemic risk being forgotten [[10], [11], [12],[14], [15], [16]]. Health security extends far beyond emergency responses [17]. It includes HCWF sustainability as a foundational pillar, as well as preparedness, resilience and protection of essential services. While investments in defence [18], biomedical innovation [7] and digital security [19] have expanded significantly, HCWF stability and resilience are not core priorities within the evolving European Health Union architecture.

This policy commentary argues that HCWF preparedness and protection must become a structural pillar of emerging EU health and security policy and an essential infrastructure for its implementation. Without an empowered HCWF with sufficient staffing levels, appropriate competencies and decent working conditions, new health security and emergency governance frameworks will not work! We highlight the urgency of a comprehensive EU policy strategy and identify capacities for implementation and co-benefits for democratic societies and economies [20,21]. Drawing on policy documents, published literature, and expert insights, the commentary proposes actionable recommendations for an EU strategy and its implementation.

## 2 What matters for preparing the health and care workforce for health security?

**Fig. 1:**
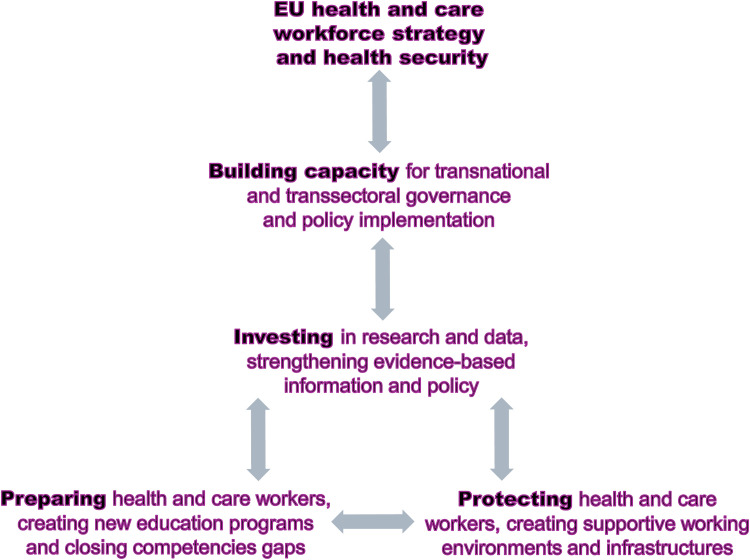
EU health and care workforce strategy and health security.

Source: authors’ own figure.

The HCWF crisis and health security are interconnected (Fig. 1), calling for a multi-level approach that systematically combines four major steps.

First, closing the competency gaps to respond to new forms of demand [6,22]. Policy priorities include preparing for civil defence and HCWF protection under military aggression [[23], [24], [25]]; identifying and preventing cyberattacks and disinformation while using AI technologies effectively and ethically [26,27]; responding to emerging infectious diseases, extreme heat waves, floods and other climate related threats [28,29]; and consolidating lessons learned from COVID-19 [10,11,15,30]. While some competencies may be highly specific, most are generic, requiring interprofessional learning, joint curricula and shared qualification standards.

Second, protecting the HCWF by improving working conditions, mental health support and violence prevention [2,14,31,32]. Appropriate staffing levels and HCW wellbeing are prerequisites for the effective use of competencies. This requires safe organisational environments, political support, legal protection and sustained public trust.

Third, investing in research and data spaces to strengthen evidence-based information and policy-making [26,33]. Reliable data and transparent evidence are essential for health literacy and for protecting HCWs and the general public against disinformation, anti-democratic and totalitarian narratives, including anti-vaccination campaigns and populist and nationalist movements [6,34].

Fourth, strengthening policy development and governance structures [12] at EU and national/regional levels [4,35]. Governance acts as the ‘glue’ connecting policy and practice and enabling transsectoral and participatory approaches [11]. It supports HCWs’ self-optimising efficacy [36], problem solving capacity, and transformational leadership [37].

## 3 Why a European union strategy and why now?

It has never been more important for the EU to demonstrate its capacity for effective problem solving. Both HCWF challenges and security threats are driven by transnational and transsectoral dynamics involving global actors, requiring collaborative international/European responses in which public health is a central pillar [18].

The HCWF also plays an important role beyond healthcare delivery. A well prepared HCWF strengthens health system resilience and builds trust in healthcare, public institutions, and democratic governance [17,34,38]. In a context of rising populisms and authoritarian movements, the EU can demonstrate its added value to member states and globally.

The EU must therefore intensify efforts towards a coherent HCWF crisis strategy [4,35] that aligns health security and workforce policies across countries, sectors and occupational groups. This is not an easy task! It will require political commitment and a strategic framework capable of guiding member states through the poly-crisis. While the challenges are considerable, they also provide an opportunity to strengthen the EU’s role within Europe and globally [5].

## 4 How to build capacities for an EU strategy and implementation?

Established governance frameworks, legal instruments, economic strength, stakeholder alliances and robust research infrastructures and funding programs provide a strong foundation for an EU HCWF strategy [4,35,39,40]. Three complementary approaches must be aligned to maximise capacities.

First, build on existing research, education and training programs, stakeholder networks and governance structures, scaling and adapting them to new demands. The EU Qualification Directive [41] could be updated to expand competencies and professional scopes to close gaps. Erasmus programs, the European Semester, the EU data space initiative, COVID-19 collaboration mechanisms, health labour market regulation and the EU Working Time Directive, alongside professional networks [42] and small country alliances [43] provide further opportunities for improving education, research and working environments for HCWs [4,16,35].

Second, and most importantly, strengthen cross sectoral co-benefits and allocate portions of existing large-scale budgets to HCWF preparedness and protection. Relevant funding streams include defence and NATO armament budgets, digital technology and cyber security programs, and pharmaceutical and biomedical innovation funding. Generating stronger evidence on co-benefits [21] could unlock resources for the HCWF across sectors, including private investment.

Third, while adapting existing policies is necessary, the poly-crisis requires deeper transformation. HCWF stability and resilience must become a core pillar of all health security programs, and health security competencies must be made an integral part of HCW education and training. Strengthening public health leadership, promoting knowledge exchange and preparing HCWs to provide care under high-risk conditions [24,25,30,44] are central to this transformation.

## 5 Conclusions

A resilient HCWF must become a structural pillar of EU health and security policy and a core infrastructure for its implementation. Without a well prepared and protected HCWF, health security will not be possible! The interconnected crisis of workforce fragility and security threats affect all European countries, calling for coordinated EU action. Although a coherent strategy and effective governance framework are still lacking, Europe can build on established programs, tools and infrastructures to prepare and protect its HCWF and strengthen health security.

**Policy recommendations**

➢ Develop a coordinated EU health security and workforce strategy to transform crisis into capacity and provide guidance for member states.
➢ Close competency gaps to ensure preparedness for military aggression, cyber threats, and climate related emergencies.
➢ Invest in research and data spaces to strengthen evidence-based policy for HCWF protection and retention as pillars of health security.
➢ Create governance structures and collaborative networks across countries, sectors and professional groups to reinforce workforce resilience.
➢ Build on existing EU governance frameworks, programs and budgets to strengthen capacities and resources for HCWF and health security development.

## Funding

This research did not receive any specific grant from funding agencies in the public, commercial sector, or non-for-profit sectors.

## CRediT authorship contribution statement

**Ellen Kuhlmann:** Conceptualization, Project administration, Writing – original draft, Writing – review & editing. **Tiago Correia:** Writing – review & editing. **Katarzyna Czabanowska:** Writing – review & editing. **Michelle Falkenbach:** Writing – review & editing. **Marius-Ionuț Ungureanu:** Writing – review & editing. **Matthias Wismar:** Writing – review & editing. **Tomas Zapata:** Writing – review & editing.

## Declaration of competing interest

None of the authors have any conflicts of interest to disclose concerning this paper.
